# HEALTH CARE USE AMONG SPOUSE CAREGIVERS OF OLDER ADULTS LIVING WITH DEMENTIA

**DOI:** 10.1093/geroni/igae098.3133

**Published:** 2024-12-31

**Authors:** Lianlian Lei, Kenneth Langa, Edward Norton, Helen Levy, Hyungjin Myra Kim, Frederic Blow, Donovan Maust

**Affiliations:** University of Michigan, Ann Arbor, Michigan, United States; University of Michigan, Ann Arbor, Michigan, United States; University of Michigan, Ann Arbor, Michigan, United States; University of Michigan, Ann Arbor, Michigan, United States; University of Michigan, Ann Arbor, Michigan, United States; University of Michigan, Ann Arbor, Michigan, United States; University of Michigan, Ann Arbor, Michigan, United States

## Abstract

Little is known about how caregiving is associated with caregivers’ health care use, particularly where the care recipient is a person living with dementia [PLWD]. This study aims to characterize health care use of spouses of PLWD. Using the 2000–2018 Health and Retirement Study linked with Medicare claims data, we identified community-dwelling dyads where one member had dementia, both members were aged ≥65 years, and enrolled in fee-for-service Medicare; for dyads included in multiple waves, we randomly selected one wave (N=924 unique dyads). We examined the association of spouse care intensity with spousal health care use, adjusted for spousal socio-demographic, health, and functional characteristics. Forty-three percent of spouses did not provide care; and among those providing care, the average monthly hours were 27, 150, and 430 for the first, second, and third tertiles, respectively. Adjusted for spousal characteristics, spouses not providing help had higher total annual Medicare costs ($5,728 and $5,776 higher than those in the second and third tertiles, respectively, p<.05 for both), acute hospitalization (8.2% and 10.4% higher than those in the second and third tertiles, respectively, p<.05 for both), and sub-acute care (7.2% higher than those in the second tertile, p<.01). The spouses who did not provide caregiving hours had the highest health care costs and use. It is unclear whether PLWD spouses do not need care or whether these spouses, by virtue of their own health problems, do not have the capacity to provide caregiving.

